# Adenoviral-Vectored Mayaro and Chikungunya Virus Vaccine Candidates Afford Partial Cross-Protection From Lethal Challenge in A129 Mouse Model

**DOI:** 10.3389/fimmu.2020.591885

**Published:** 2020-11-04

**Authors:** Rafael Kroon Campos, Lorena Preciado-Llanes, Sasha R. Azar, Young Chan Kim, Olivia Brandon, César López-Camacho, Arturo Reyes-Sandoval, Shannan L. Rossi

**Affiliations:** ^1^ Department of Microbiology and Immunology, University of Texas Medical Branch, Galveston, TX, United States; ^2^ Nuffield Department of Medicine, The Jenner Institute, University of Oxford, Oxford, United Kingdom; ^3^ Department of Pathology, University of Texas Medical Branch, Galveston, TX, United States; ^4^ Institute for Human Infection and Immunity, University of Texas Medical Branch, Galveston, TX, United States

**Keywords:** ****adenovirus-vectored vaccines, alphavirus, chikungunya virus, cross-protection, arthritis, A129 mice, Mayaro virus, chimpanzee adenovirus

## Abstract

Mayaro (MAYV) and chikungunya viruses (CHIKV) are vector-borne arthritogenic alphaviruses that cause acute febrile illnesses. CHIKV is widespread and has recently caused large urban outbreaks, whereas the distribution of MAYV is restricted to tropical areas in South America with small and sporadic outbreaks. Because MAYV and CHIKV are closely related and have high amino acid similarity, we investigated whether vaccination against one could provide cross-protection against the other. We vaccinated A129 mice (IFNAR −/−) with vaccines based on chimpanzee adenoviral vectors encoding the structural proteins of either MAYV or CHIKV. ChAdOx1 May is a novel vaccine against MAYV, whereas ChAdOx1 Chik is a vaccine against CHIKV already undergoing early phase I clinical trials. We demonstrate that ChAdOx1 May was able to afford full protection against MAYV challenge in mice, with most samples yielding neutralizing PRNT_80_ antibody titers of 1:258. ChAdOx1 May also provided partial cross-protection against CHIKV, with protection being assessed using the following parameters: survival, weight loss, foot swelling and viremia. Reciprocally, ChAdOx1 Chik vaccination reduced MAYV viral load, as well as morbidity and lethality caused by this virus, but did not protect against foot swelling. The cross-protection observed is likely to be, at least in part, secondary to cross-neutralizing antibodies induced by both vaccines. In summary, our findings suggest that ChAdOx1 Chik and ChAdOx1 May vaccines are not only efficacious against CHIKV and MAYV, respectively, but also afford partial heterologous cross-protection.

## Introduction

Mayaro virus (MAYV) and chikungunya virus (CHIKV) are arboviruses, members of the *Togaviridae* family, and the etiologic agents of Mayaro fever (MAYF) and chikungunya fever (CHIKF), respectively. Both illnesses are characterized by flu-like symptoms including fever, myalgia, arthralgia and/or skin rash (1–4), making their symptomatology largely indistinguishable from each other and from other common arboviral diseases (5–7). CHIKV circulates in many continents (8), whereas MAYV is thought to be restricted to areas close to forests in Central and South America, where it causes small outbreaks (9–13). However, since MAYV is present in regions where many arboviruses co-circulate, the number of human infections is likely underreported. There is co-incidence of both diseases in the Americas, especially in South America, where it is estimated that 1% of all febrile cases with symptoms and clinical signs of arboviral disease may be caused by MAYV (7). Although most outbreaks have been small, its potential to produce large outbreaks became evident in 1978, when MAYV was responsible for infecting approximately 20% of the 4,000 inhabitants in Belterra, Brazil, most living near the forest (12). Although MAYV is able to cause disease in humans and produce high viremia, mosquitoes of the *Haemagogus* genus, which are the primary vectors of MAYV, are absent in urban settings (6, 14). Vector competency studies in laboratory settings have reported that MAYV may be transmitted by urban and peri-urban mosquitoes of the *Aedes* genus (15–17). Although MAYV has been isolated from *Aedes aegypti* in nature (18), transmission from these mosquitoes to humans has not been reported to date. MAYV could adapt to emerge into an urban transmission cycle, just as was determined to have happened for its close relative CHIKV, which adapted to *Aedes albopictus* after acquiring a mutation in the amino acid in the position 226 of the E1 viral protein (19, 20). Due to the presence of both viruses in the same regions, and the possibility of MAYV adaptation to the urban cycle (13, 21), there is significant interest in developing vaccines which could simultaneously protect against both diseases. Therefore, it is important to understand the impact that vaccination for CHIKV may have on MAYF and reciprocally, the effect that vaccination for MAYV may have on CHIKF. The similarities between MAYV and CHIKV are vast, not only in their mode of transmission and disease profile, but also in their viral structure and antigenic relationship. Thus, it is not surprising that several studies have investigated the possibility of cross-protection between MAYV, CHIKV and other alphaviruses (22–25). Webb and colleagues (25) reported different degrees of protection with two CHIKV candidate vaccines. The live-attenuated vaccine CHIKV-IRES, protected partially against MAYV challenge, whereas the chimeric host-restricted vaccine EILV-CHIKV did not protect against MAYV disease.

As CHIKV has a noteworthy health and economic burdens and MAYV may emerge to pose serious threats, it is imperative that countermeasures are developed to prepare against outbreaks, however, no licensed vaccine is available to date. Several strategies have been used to develop vaccines against alphaviruses, including live-attenuated virus, viral protein subunits, viral vectors and nucleic acid-derived vaccines (26, 27). Engineered adenoviral-vectored vaccines have been widely investigated (28, 29), as they are known to be potently immunogenic, inducing both antibodies and T cell responses. However, the use of human adenoviruses has been limited, mainly due to pre-existing immunity against these viruses among the general population (30). To circumvent this issue, chimpanzee adenoviruses are being used due to their negligible seroprevalence in human populations (30, 31). ChAdOx1 is a chimpanzee adenoviral vector, developed from the adenovirus isolate Y25 subgroup E (32). ChAdOx1 has deletions on the E1 and E3 genes that render it replication-deficient thereby enhancing its safety (32). We have previously reported that ChAdOx1 encoding CHIKV structural proteins (ChAdOx1 Chik) elicits long-lasting IgG antibodies against CHIKV E2 in BALB/c mice (33). ELISA measurements at two weeks, six weeks and 10 months post vaccination, showed that the levels of IgG anti-CHIKV E2 are maintained over time, suggesting long term immunity of at least 10 months. In the same work, we also found a high frequency of T cells recognizing CHIKV peptides as early as two weeks after immunization, thereby suggesting that ChAdOx1 Chik induces specific T cell responses (33). In another study, we found that ChAdOx1 Chik provides complete protection from a lethal CHIKV challenge in the highly permissive A129 mouse model (34).

We have constructed ChAdOx1 May, a chimpanzee adenoviral vectored vaccine that expresses the MAYV structural proteins. In this study, we demonstrate that ChAdOx1 May elicits rapid and robust immunity with high titers of neutralizing antibodies against MAYV, able to protect A129 mice from a lethal and reducing viremia to undetectable levels. Furthermore, we show that vaccination with ChAdOx1 May offers cross-protection against a lethal CHIKV challenge. Conversely, the equivalent chikungunya vaccine named ChAdOx1 Chik, and which is currently undergoing clinical trials (NCT03590392), appears to have a very limited effect against MAYV. Our results are particularly relevant in the setting of outbreaks, where pre-existing immunity against MAYV may lead to immunity against CHIKV, and vice versa.

## Materials and Methods

### Design and Production of the ChAdOx1 May Vaccine

The structural cassette MAYV sequence derived from various MAYV lineages was codon optimized. To improve initiation of translation a Kozak consensus sequence was included before the 5’ end of the transgene. Finally, the transgene design included the required enzymatic restriction sites to allow the in-frame cloning of the transgene between the CMV promoter and the PolyA sequence region contained in our shuttle and expression vector (pMono). A synthetic gene cassette was produced by GeneArt^®^ (Fisher Scientific, Regensburg, Germany) and was named sMAYV. The plasmid containing the structural Mayaro virus (sMAYV) cassette (Capsid, Envelope 1–3 and 6K) was digested with KpnI and NotI restriction enzymes (NEB, Ipswich, MA, U.S.) to allow in-frame ligation between the CMV promoter and the Poly(A) regions contained in the shuttle plasmid (pMono). The recombinant DNA plasmids were expanded and purified from *E. coli* using the Qiagen MIDI-prep kit. Resulting plasmids were verified by restriction analysis and 5’ and 3’ flanking sequencing. To generate ChAdOx1 vaccine, the shuttle plasmids containing attL regions sequences were each recombined with those attR regions contained in the destination vector ChAdOx1 using an *in vitro* Gateway reaction (LR Clonase II system, Invitrogen™). Successfully recombined ChAdOx1 May (also known as ChAdOx1 sMAYV) was verified by DNA sequencing using flanking primers (forward promoter primer and Poly-(A) reverse primer). Standard cell biology and virology techniques were performed to generate the non-replicative adenoviral vectors.

### Design and Production of the ChAdOx1 Chik Vaccine

ChAdOx1 Chik (also known as ChAdOx1 sCHIKV) was designed and produced as previously reported (33). The immunogenicity and efficacy profiles of ChAdOx1 in mice has been recently demonstrated (33, 34).

### Control Vaccines

ChAdOx1 Zika (also known as ChAdOx1 Zika prME ΔTM) was produced as previously described (35) and used as the off-target control vaccine in our challenge experiments. The MAYV-IRES vaccine were previously developed (36), by inserting an IRES in the genome of MAYV and passaging, respectively. Unrelated ChAdOx1 dengue NS1 (unpublished) was used as the mock vaccine in the immunogenicity studies.

### Viruses and Cells Used

Vero CCL-81 cells from the American Type Culture Collection were grown in Dulbecco’s modified Eagle medium (DMEM) containing 10% of fetal bovine serum (FBS) and 1% of penicillin/streptomycin at 37˚C in a humidified incubator containing 5% of CO_2_. The MAYV-IRES cDNA clone was used to produce viral stocks by electroporating Vero cells as previously reported (36, 37).

### Virus Titration

Samples underwent 10-fold serial dilutions in DMEM with 2% FBS and incubated on monolayers of Vero cells as described previously. After 1 h of incubation at 37°C rocking every 15 min, an 0.4% agarose overlay was added. Cells were then incubated at 37°C with a 5% CO_2_ atmosphere for 48h (for MAYV) and 36h (for CHIKV), fixed with a solution of 3.7% formaldehyde and stained with crystal violet (0.25% w/v in 30% methanol). Titers were shown as PFU/ml and had a limit of detection (LOD) of 100 PFU/ml. In the statistical analyses, values below LOD were set as 50% of the LOD (50 PFU/ml).

### Plaque Neutralization Reduction Test

PRNT assays were done using the same viruses used in the challenges, MAYV CH strain (also known as IQT4235 strain) and CHIKV La Reunion (LR) strain, as previously described. Sera was heat-inactivated for 1 h at 56°C and underwent 2-fold serial dilutions in media. The virus was then incubated with the serum for 1h at 37°C and then added to monolayers of Vero cells and then treated as a virus titration from that step. A reduction of 50% or 80% in the number of virus plaques compared to virus only control was used to call PRNT50 and PRNT80 cutoffs, respectively. The LOD was 1:20.

### Animals

The A129 mice were purpose-bred from a colony maintained at UTMB, which is an AALAS-approved facility. Mice were kept in sterilized cages. Cohorts with male and female mice were ear-notch identified and vaccinated at 5 weeks-old. ChAdOx1 vaccines were diluted to deliver a dose of 1 x 10^8^ IU and MAYV-IRES vaccine was diluted in PBS and the inoculum was backtitered to be 1.6 x 10^4^ PFU/mouse. For each mouse, 25 µl of vaccine was injected intramuscularly in each leg. All the work was done according to our approved Institutional Animal Care and Use (IACUC) protocol (1708051). Mice reaching a humane endpoint such as a loss of 20% or greater of their body weight or any evidence of neurological disease (including inability to move when stimulated, inability to eat/drink, tremors and paralysis) were euthanized by CO_2_ asphyxiation. The number of animals used for each condition are indicated in **Table 1**.

**Table 1:** Survival of A129 mice challenged with Mayaro virus *(*MAYV) or chikungunya virus (CHIKV).

Vaccine	Challenge	Number of mice in cohort	% Survival (number)	MDD^1^	*p*-value^2^
PBS	MAYV	6	0% (0/6)	3 +/− 0	N/A
ChAdOx1 Zika	MAYV	5	0% (0/5)	3.6 +/− 0.5	0.0339
MAYV-IRES	MAYV	5	100% (5/5)	N/A	0.0016
ChAdOx1 May	MAYV	5	100% (5/5)	N/A	0.0016
ChAdOx1 Chik	MAYV	5	60% (3/5)	9.5 +/− 3.5	0.0016
PBS	CHIKV	4	0% (0/4)	4.4 +/− 0.5	N/A
ChAdOx1 Zika	CHIKV	5	0% (0/5)	4.4 +/− 0.5	0.1763
ChAdOx1 Chik	CHIKV	5	100% (5/5)	N/A	0.0047
ChAdOx1 May	CHIKV	5	80% (4/5)	9 +/- 0	0.0047

^1^Mean Day of Death (MDD) +/− standard deviation. Excludes mice that survived.

^2^p-value, based upon Log-ranked (Mantel-Cox) test compared with PBS.

Female inbred BALB/c (H-2d), (6–8 weeks) were used for the assessment of immunogenicity (n = 6 per group). Mice were purchased from Envigo RMS Inc. (Bicester, G.B.). The experimental design took into account the 3R reduction (Replacement, Reduction, Refinement) and procedures were approved by the Animal Care and Ethical Review Committee (PPL 30/2414). No randomization was used in this work.

### Vaccination

ChAdOx1 vaccines were thawed on ice and MAYV-IRES at 37°C. All vaccines were then diluted in Dulbecco’s phosphate buffered saline (DPBS). ChAdOx1 vaccines were administered at 1 × 10^8^ infectious units (IU) per animal. MAYV-IRES was titrated after vaccination and determined to be 1.3 x 10^4^ PFU/mouse. Animals were anesthetized using isoflurane and then injected with 25 µl of vaccine intramuscularly in each hind leg.

### Challenge and Monitoring of Morbidity Readouts

Mice were challenged thirty days post-vaccination. The challenge viruses used were MAYV-CH (backtiter: 1.6 x 10^4^ PFU/mouse) and CHIKV-LR (backtiter: 9.7 x 10^4^ PFU/mouse). Mice were anesthetized with isoflurane and MAYV-CH or CHIKV-LR were injected intradermally in a volume of 20 µl of virus into the left foot with a 28G insulin syringe. Back titration of the challenge viruses was 1.6 x 10^4^ and 9.7 × 10^4^ and PFU/mouse for MAYV and CHIKV, respectively. After injection, mice health and weights were monitored daily and mice that lost more than 20% of their starting weight were euthanized. Footpad thickness was also measured daily after infection as previously reported (34) and mice were provided with soft bedding and nesting materials to reduce stress and pain. On day 25 post vaccination and day 2 post infection, blood was collected from anesthetized mice using a capillary tube on the retro-orbital sinus. Blood and sera collected from clarified blood samples were used for PRNT assays and viremia tests, respectively.

### Enzyme-Linked Immunosorbent Assay

Specific antibody binding to MAYV or CHIKV envelope proteins (E2 or E1) was measured by an IgG enzyme linked immunosorbent assay (ELISA) as previously described (33). Briefly, mice sera were diluted in Nunc Maxisorp Immuno ELISA plates coated with the MAYV or CHIKV envelope proteins (E2 or E1) diluted in PBS to a final concentration of 5 µg/mL and incubated at room temperature (RT) overnight. Plates were washed 6 times with PBS/0.05% Tween (PBS/T) and blocked with 300 µL with Pierce™ protein-free (PBS) blocking buffer (Thermo Fisher Scientific, Waltham, MA, U.S.) for 2 h at RT. Mouse serum was added and serially diluted 3-fold down in PBS/T with 50 µL per well as final volume and incubated for 2 h at RT. Following washing 6 times with PBS/T, bound antibodies were detected following a 1 h incubation with 50 µL of alkaline phosphatase-conjugated antibodies specific for whole mouse IgG (A3562-5ML, Sigma Aldrich, SLM, U.S.). Following an additional 6 washes with PBS/T, development was achieved using 100 µL of 4-nitrophenylphosphate diluted in diethanolamine buffer and the absorbance values at OD405 were measured and analyzed using a CLARIOstar instrument (BMG Labtech, Aylesbury, GB). Serum antibody endpoint titers were defined by an absorbance value three standard deviations greater than the average OD_405_ of the control.

### Production of MAYV Proteins for ELISA

For expression and purification of the MAYV E2 protein, the codon-optimized gene of E2 (a.a. 1–351) was cloned into the pHLsec vector. In order to improve secretion of the E2 protein, the C-terminal region of E2 (a.a. 352–422) was removed. The pHLsec MAYV E2 plasmid (500 µg) was transfected in HEK-293T cells using polyethyleneimine (PEI) in roller bottles (surface area of 2,125 cm2) under standard cell culture conditions. Five days after transfection cells were discarded and media was filtered through 0.22 µM disposable filters. The secreted protein was purified from the supernatant by Ni Sepharose affinity chromatography (HisTRAP™, GE Healthcare), using the Äkta Start chromatography system and eluted with Imidazole 500mM. Finally, the eluted protein was dialyzed using Slide-A-LyzerTM cassette against 1X PBS. The MAYV E1 and CHIKV E1 proteins were produced in a similar manner using the codon-optimized genes of CHIKV and MAYV E1 (a.a 1-410). CHIKV E2 protein was produced as previously described (33).

### Statistical Analyses

Statistical analysis was performed in GraphPad Prism v8.4. Data was analyzed by one-way ANOVA, two-way ANOVA or restricted maximum likelihood (REML) mixed model as appropriate. *Post hoc* corrections were implemented with Dunnett`s against a control group or Sidak’s when comparing selected groups. Virus titer data were log_10_ transformed before statistical analyses. Survival curve comparisons were made using a log-ranked (Matel-Cox) test. In all statistical tests *p* values below 0.05 were considered significant.

## Results

### ChAdOx1 May Induces Antibodies Against MAYV E2 and May E1 as Early as 2 Weeks Post-Vaccination in BALB/c Mice

**Figure 1:**
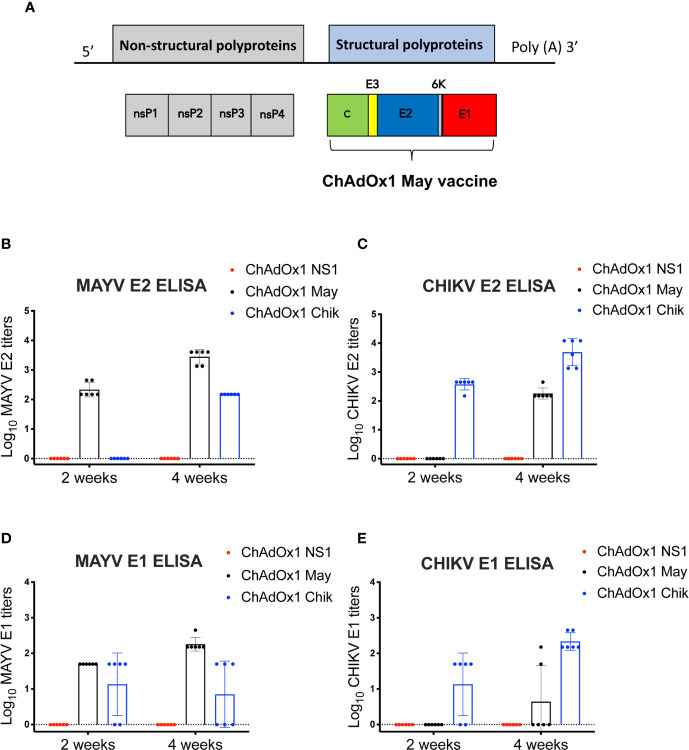
Mayaro virus (MAYV) genome organization, design of ChAdOx1 May vaccine candidate and the humoral responses elicited. **(A)**. Genome organization of MAYV and generation of ChAdOx1 May vaccine. **(B–E)**. Humoral IgG responses against chikungunya virus (CHIKV) and MAYV E1,E2 2 and 4 weeks post-immunization measured by ELISA. The reciprocal log ELISA titers were calculated for all groups shown in the figure. Lines represent the mean with SD and error bars are shown.

We constructed ChAdOx1 May, a chimpanzee adenoviral vectored-vaccine that expresses the MAYV structural proteins capsid, E1, E2, E3, and 6K (**Figure 1A**). To assess the specific immunogenicity of ChAdOx1 May vaccine, we immunized groups of BALB/c mice (n=6) with a single and unadjuvanted dose of ChAdOx1 May or ChAdOx1 Chik at 1 × 10^8^ infectious units (IU) per animal. Specific IgG antibody responses against E1 or E2 proteins from both MAYV and CHIKV were measured by ELISA at 2 weeks and 4 weeks after immunization.

At 2 weeks post-immunization, the mean MAYV E2-specific antibody titers elicited by ChAdOx1 May was 2.34 log_10_, whereas the mean CHIKV E2-specific antibody titers elicited by ChAdOx1 Chik was 2.57 log_10_. By week 4, the anti-E2 titers for both ChAdOx1 May and ChAdOx1 Chik vaccinated animals increased to 3.45 log_10_ and 3.69 log_10_, respectively (**Figures 1B, C**). No specific IgG antibody binding to MAYV E2 or CHIKV E2 was detected in the mock-vaccinated group (ChAdOx1 dengue NS1) at any time point. No cross-reactive IgG antibody binding to MAYV E2 or CHIKV E2 was detected at 2 weeks. However, at 4 weeks after vaccination, anti-MAYV E2 antibodies were detected in mice vaccinated with ChAdOx1 Chik (mean titers 2.18 log_10_). ChAdOx1 May vaccinated animals also showed cross-reactive anti-CHIKV E2 antibodies (mean titer 2.26 log_10_) by week 4. This indicates that there is some degree of cross-reactivity between anti-CHIKV E2 antibodies toward the MAYV E2 protein and vice versa.

ChAdOx1 May vaccinated mice had a mean anti-MAYV E1 antibody titer of 1.70 log_10_ at 2 weeks post-immunization and this increased to 2.26 log_10_ by 4 weeks (**Figure 1D**). Four and three mice vaccinated with ChAdOx1 Chik had detectable cross-reactive anti-MAYV E1 antibodies at 2 weeks and 4 weeks after vaccination, respectively. Vaccination with ChAdOx1 Chik induced anti-CHIKV E1 antibodies in four out of six animals at 2 weeks, and by 4 weeks all mice had detectable anti-CHIKV E1 antibodies (mean titer 2.33 log_10_) (**Figure 1E**). Mice vaccinated with ChAdOx1 May did not show any cross-reactive anti-CHIKV E1 antibodies at 2 weeks post-immunization, but two mice had detectable anti-CHIKV E1 antibodies at 4 weeks.

Taken together, we show that a single dose of ChAdOx1 May and ChAdOx1 Chik is immunogenic and induces specific anti-E1 and E2 IgG antibodies as early as two weeks post-immunization. Moreover, our results suggest that ChAdOx1 May and ChAdOx1 Chik induce cross-reactive antibodies, in particular toward the E2 protein at 4 weeks post-immunization.

### ChAdOx1 Vaccination Do Not Cause Adverse Events in A129 Mice

**Figure 2:**
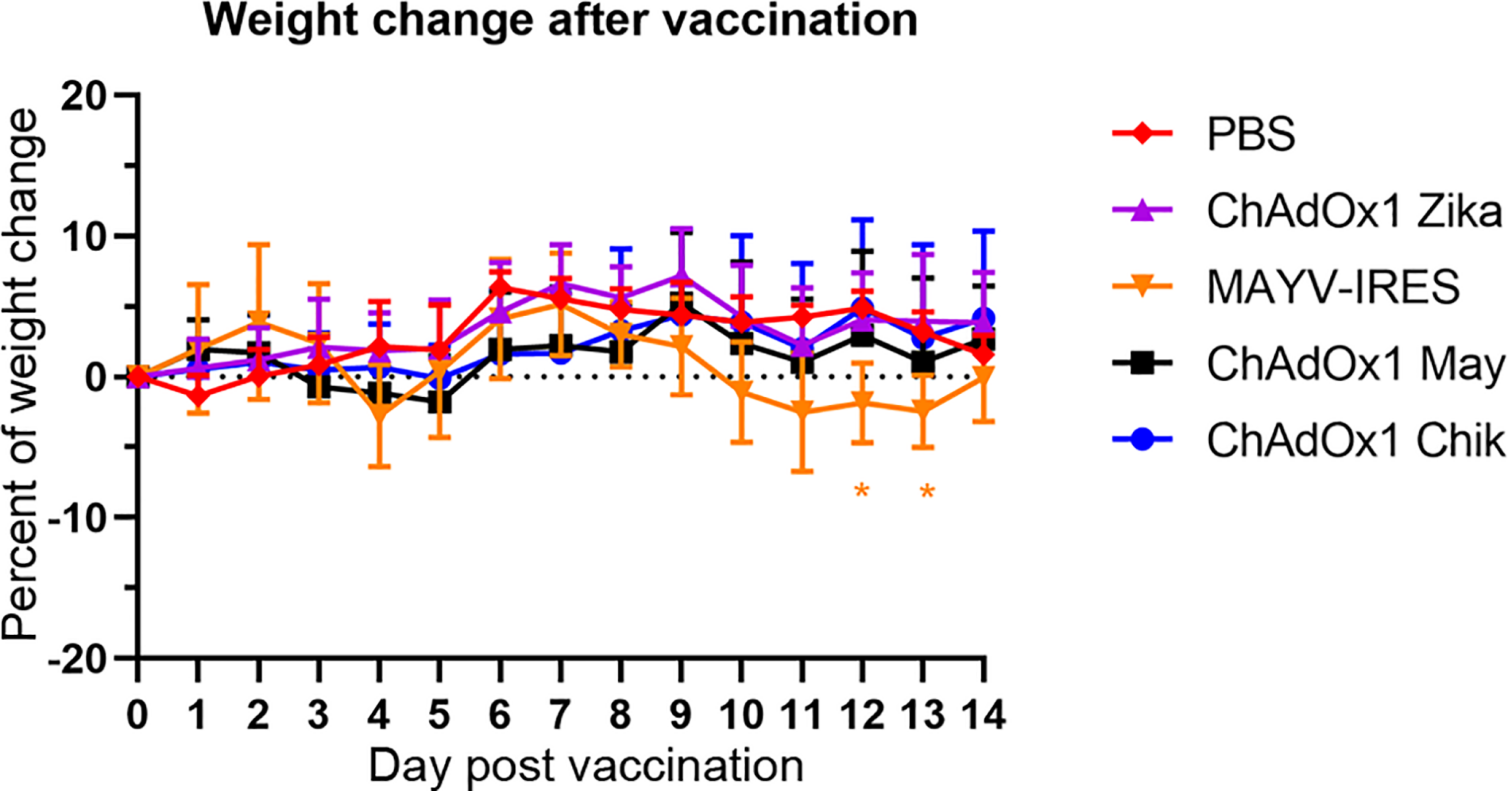
Administration of ChAdOx1 vaccines do not cause weight loss in A129 mice. Percentages of weight change following vaccinations are shown. The weights of A129 mice in each group were compared to their weights just before vaccination (day 0). Data are represented as means and SEM. Two-way ANOVA with repeated measures with Dunnet*’*s compared with PBS group; *p < 0.05.

Next, we sought to determine if the antibody response observed in vaccinated BALB/c mice would elicit protective immunity in the A129 mouse challenge model. A129 mice that are deficient in IFN-α/β receptor signaling pathway offer a rigorous test for vaccine safety because interferon is important for an efficient antiviral response, and as such, A129 mice are highly susceptible to infections and lethal disease. In agreement with our previous publication (34), we did not observe weight loss or any adverse events in A129 mice (IFNAR −/−) vaccinated with any of the ChAdOx1 viral vectors, including ChAdOx1 Chik, ChAdOx1 May or the off-target vaccine (ChAdOx1 Zika, **Figure 2**). In contrast, the live-attenuated MAYV-IRES vaccine used as a positive control (36), caused adverse clinical signs such as mild weight loss (**Figure 2**), lethargy, ruffled fur and squinty eyes. MAYV-IRES vaccinated animals recovered completely prior to challenge, except for one mouse that continued presenting squinty eyes until the end of the experiment, a sequela likely caused by MAYV-IRES.

### ChAdOx1 May and ChAdOx1 Chik Afford Homologous Protection and Partial Heterologous Cross-Protection Against MAYV and CHIKV-Induced Disease in A129 Mice

To test the effectiveness of our ChAdOx1 May and ChAdOx1 Chik candidate vaccines, and to investigate whether they could induce cross-protection, we carried out CHIKV and MAYV challenges in A129 mice. Thirty days following vaccination, mice were challenged with a lethal dose of MAYV-CH (1.6 x 10^4^ PFU/mouse) or CHIKV-LR (9.7 x 10^4^ PFU/mouse), *via* intradermal injection on the left rear foot. Survival, weight loss, foot swelling, and other signs associated with MAYV or CHIKV-induced disease were assessed daily and used as readouts.

We recently demonstrated that vaccination with ChAdOx1 Chik prevents lethal disease in mice when challenged with CHIKV (34). Here, we demonstrate that mice vaccinated with ChAdOx1 May and the live-attenuated MAYV-IRES control vaccine were protected and all survived MAYV challenge until the end of the experiment (**Table 1**). Remarkably, ChAdOx1 May and ChAdOx1 Chik vaccines demonstrated different degrees of cross-protection, three out of five mice vaccinated with ChAdOx1 Chik survived the MAYV challenge, whereas four out of five mice vaccinated with ChAdOx1 May survived the CHIKV challenge (**Table 1**). As expected, mice vaccinated with PBS or ChAdOx1 Zika were not protected against MAYV or CHIKV infections and had to be euthanized a few days after challenge.

**Figure 3:**
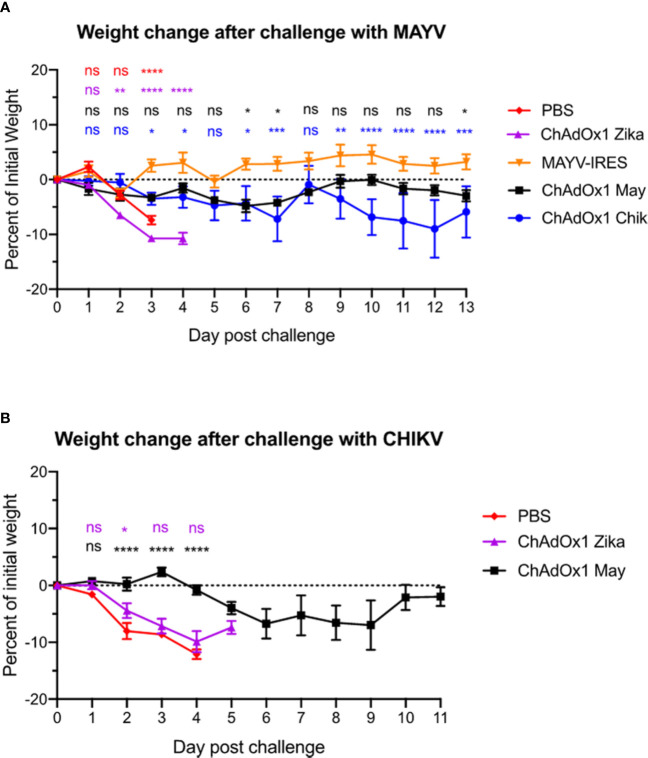
ChAdOx1 May and ChAdOx1 Chik vaccination protect against weight loss. Animals were challenged thirty days post-vaccinations with Mayaro virus (MAYV) or chikungunya virus (CHIKV) (backtiters 1.6 and 9.7 x 10^4^ PFU/mouse). **(A)** Weight change in vaccinated mice after challenge with MAYV. Data represented as mean and SEM, restricted maximum likelihood mixed model with Dunnett’s (compared with MAYV-IRES group). **(B)** Weight change in vaccinated mice after challenge with CHIKV. Data represented as mean and SEM, restricted maximum likelihood mixed model with Dunnett’s (compared with PBS group) *p < 0.05, **p < 0.01, ***p < 0.001, ****p < 0.0001. ns, not significant.

In comparison with the control groups injected with PBS and the ChAdOx1 Zika vaccine, which lost about 10% of their weight by day 3 after MAYV challenge, both MAYV-IRES and ChAdOx1 May vaccines protected mice against significant weight loss (**Figure 3A**). ChAdOx1 Chik provided partial cross-protection by preventing significant weight loss in two out of five animals (40%) and by delaying weight loss in 1/5 mice (**Figure 3A**). In an equivalent CHIKV challenge model, we previously demonstrated that ChAdOx1 Chik protected mice against weight loss, viremia and foot swelling at the inoculation site (34). In this CHIKV challenge, vaccination with ChAdOx1 May resulted in a delayed and mild weight loss, with only one out of five mice (20%) in the group losing over 20% of its weight by day 9 (**Figure 3B**).

**Figure 4:**
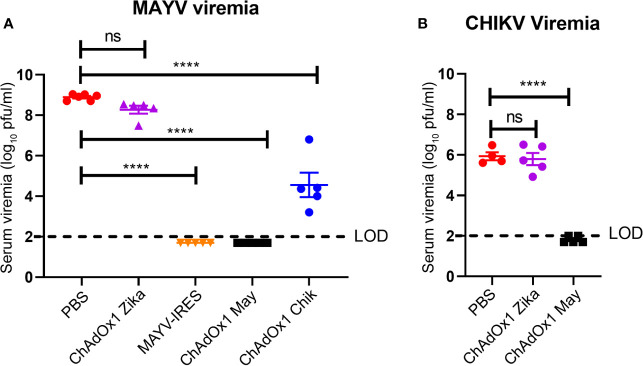
ChAdOx1 May and ChAdOx1 Chik vaccines afford cross-protection by reducing viremia. Blood was collected 2 days post challenge and serum was titrated. **(A)** Mayaro virus (MAYV) viremia. **(B)** Chikungunya virus (CHIKV) viremia. The limit of detection (LOD) is 2 log_10_ PFU/ml. Data represented as mean and SEM, one-way ANOVA with Dunnett’s, ****p < 0.0001. ns, not significant.

Viremia is an important hallmark of disease for alphavirus infections (38, 39). MAYV and CHIKV were measured in serum on the second day after challenge, when peak viremia is predicted (25, 40). Virus titers in the PBS-injected and ChAdOx1 Zika-vaccinated groups were of around 9 log_10_ PFU/ml for MAYV and around 6 log_10_ PFU/ml for CHIKV. Vaccination with ChAdOx1 May afforded sterile protection, with undetectable virus titers of MAYV in serum (**Figure 4A**). Consistent with the survival results, we observed that ChAdOx1 May and ChAdOx1 Chik provide effective cross-protection by significantly decreasing viremia. ChAdOx1 Chik vaccinated mice had on average over 3 log_10_-fold reduction in MAYV viremia compared with negative controls (**Figure 4A**). ChAdOx1 May had an even larger impact on cross-reactivity in mice following challenge with CHIKV, reducing titers by 4 log_10_-fold, with three out of five samples below the limit of detection of our assay (**Figure 4B**).

**Figure 5:**
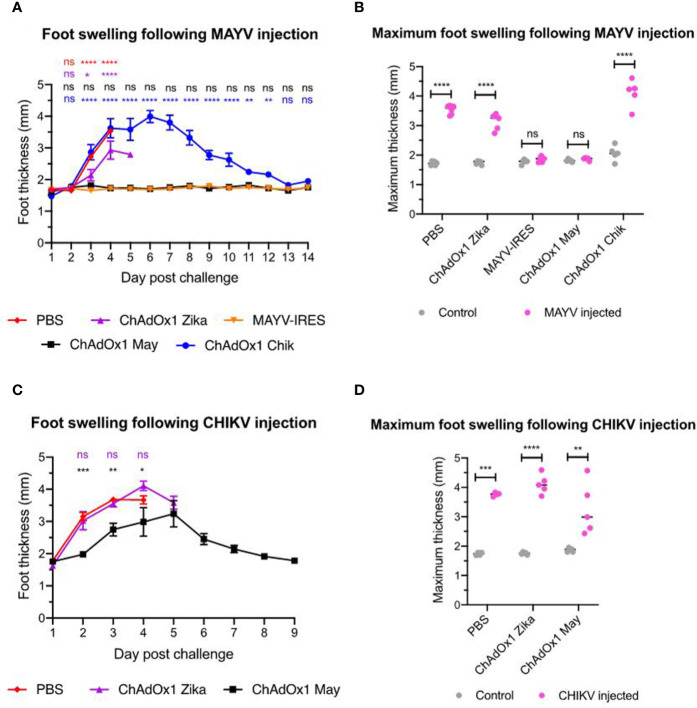
ChAdOx1 May but not ChAdOx1 Chik provides some cross-protection against foot swelling. **(A)** Foot thickness (in mm) at the Mayaro virus (MAYV) injection site. Data represented as mean and SEM, restricted maximum likelihood mixed model with Dunnett’s (compared with MAYV-IRES group). **(B)** Comparison between control foot (in grey) and the maximum foot thickness (in pink) at any given timepoint following MAYV injection. Dots represent each mouse, two-way ANOVA with Sidak’s. **(C)** Data represented as mean and SEM, restricted maximum likelihood mixed model with Dunnett’s (compared with PBS group). **(D)** Comparison between control foot (in grey) and the maximum footpad thickness (in pink) at any given timepoint following CHIKV injection. Dots represent each mouse, two-way ANOVA with Sidak’s. *p < 0.05, **p < 0.01, ***p < 0.001, ****p < 0.0001. ns, not significant.

Swelling at the inoculation site is another hallmark of arthritogenic alphavirus infection in the A129 mouse model (41), which reproduces the joint inflammation caused by MAYV and CHIKV in humans (42). In this experiment we inoculated one foot only and used the other foot as an internal control for each animal. Mice that were vaccinated with PBS or ChAdOx1 Zika showed severe foot swelling in comparison to the uninfected foot. In most of these animals, the inoculated feet quickly doubled in thickness, as early as day 3 after CHIKV inoculation and by day 4 after MAYV inoculation (**Figure 5**). Throughout the whole duration of the experiment, we did not observe foot swelling in any of the ChAdOx1 May or MAYV-IRES vaccinated mice when challenged with MAYV (**Figures 5A, B**). While vaccination with ChAdOx1 Chik failed to afford significant cross-protection in mice challenged with MAYV (**Figures 5A, B**), vaccination with ChAdOx1 May resulted in a limited but significant reduction of foot swelling in mice that were challenged with CHIKV (**Figures 5C, D**).

In summary, these results demonstrate that ChAdOx1 May not only fully protects mice from lethal MAYV-induced disease but also cross-protects against CHIKV viremia, limits CHIKV-induced weight loss, delays foot inflammation and prompts its resolution. Conversely, ChAdOx1 Chik vaccine is not as effective in cross-protecting against MAYV viremia and fails to reduce or delay foot inflammation.

### ChAdOx1 May and ChAdOx1 Chik Induce Low Levels of Cross-Neutralizing Antibodies

**Figure 6:**
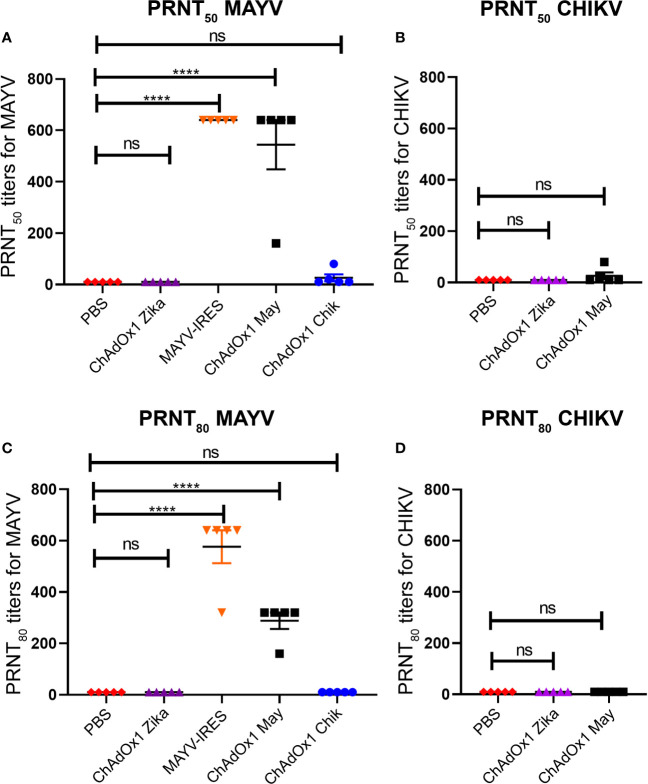
ChAdOx1 May induces high levels of neutralizing antibodies against Mayaro virus (MAYV) but low levels of cross-neutralizing antibodies. Reciprocal neutralizing antibody titers in PRNT_50_ **(A)** and PRNT_80_ **(C)** against MAYV. Reciprocal neutralizing antibody titers in PRNT_50_ **(B)** and PRNT_80_ **(D)** against CHIKV, done in a separate experiment. The limit of detection (LOD) is 20 (1:20 dilution), data points recorded as 10 had neutralization values < LOD. Dots represent titers for each animal, bars represent mean and SEM, one-way ANOVA with Dunnett’s, ****p < 0.0001. ns, not significant.

To determine whether the efficacy of the ChAdOx1 vaccines was correlated to the production of neutralizing antibodies, we performed plaque reduction neutralizing tests (PRNT) against both alphavirus using mouse sera obtained 25 days following vaccination (**Figures 6A–D**).

At the most stringent 80% cutoff (PRNT_80_) MAYV-IRES induced neutralizing titers of between 1:320 and 1:620 against MAYV, with all animals surpassing titers of 1:640 at the 50% cutoff (PRNT_50_). Sera from ChAdOx1 May vaccinated mice was also highly neutralizing against MAYV, with antibody titers ranging from 1:160 to 1:320 at PRNT_80_ and from 1:160 to 1:640 at PRNT_50_ at the vaccine dose used (**Figures 6A, C**). Only two mice from the ChAdOx1 Chik vaccinated group had detectable cross-neutralizing antibodies against MAYV at PRNT_50_, but these titers fell below the detection limit when PRNT_80_ was considered (**Figures 6A, C**).

In a separate experiment, we also measured cross-neutralizing antibodies against CHIKV in mice sera 25 days post vaccination This group had PRNT_80_ titers below the detection limit (**Figure 6D**), with low PRNT_50_ titers of 1:20 and 1:40 being detected in 2 mice (**Figure 6B**). As expected, mice vaccinated with PBS or ChAdOx1 Zika had antibody levels below our detection limit (at a 1:20 dilution) in all the PRNT performed.

## Discussion

MAYV and CHIKV are arthritogenic mosquito-borne viruses of medical importance, mainly due to the long-term polyarthritis that they can cause. CHIKV is a global threat and has caused large urban outbreaks (8), whereas MAYV has potential for emergence due to its potential to spread from a rural sylvatic cycle to an urban one (6, 12, 15). Although significant effort has been made toward vaccine development (36, 43, 44), vaccines are not yet available against these viruses. Given that MAYV and CHIKV co-circulate in Central, South America and the Caribbean and have a close phylogenic and antigenic relationship, it is imperative to evaluate not only the efficacy of candidate vaccines but also their cross-reactivity capacity.

In this study, we demonstrate that ChAdOx1 May, a novel chimpanzee adenoviral-vectored vaccine candidate, induces sterilizing immunity and high titers of neutralizing antibodies that protect A129 mice from lethal MAYV disease. We did not detect viremia in ChAdOx1 May vaccinated mice, nor observe weight loss or foot swelling which are hallmark signs of morbidity in the A129 model. We also provide evidence that ChAdOx1 May affords a good degree of cross-protection against CHIKV, by reducing lethality, preventing viremia, as well as limiting and delaying morbidity. Although cross-reactivity induced by ChAdOx1 Chik against MAYV was also observed in our model, the magnitude of the response appears variable and transitory.

Viremia is one of the hallmarks of the acute phase in alphavirus infections (45) and is considered an important factor related to the spread of these viruses by mosquitoes. As the bloodmeals taken by mosquitoes are less than 5 µl in volume (46), high titers of virus are important for them to become infected and function as a vector the virus. The effect that ChAdOx1 May had on viremia was robust and cross-reactive, lowering both MAYV and CHIKV titers to undetectable levels in our challenge model. Although we have evidence that ChAdOx1 Chik also provides sterile immunity against CHIKV (34), ChAdOx1 Chik afforded only partial cross-protection against MAYV, as reflected by a reduction in viremia by about 4 log_10_. This could be a consequence of different viral loads because, consistent with other studies, we observed that A129 mice challenged with MAYV have higher viremia at two days post infection than the titers observed in an equivalent CHIKV challenge. Reports suggest that CHIKV viremia in humans are in the magnitude of about 7 log_10_ (44), which is more than the 5.34 log_10_ titers detected in the blood of a patient with acute febrile MAYV infection (15), although the investigation on ranges of MAYV viremia has been more limited.

In mouse models used to investigate arthritogenic alphaviruses, foot swelling at the site of injection is commonly used as a readout and hallmark of morbidity (47), which reflects some of the joint inflammation that takes place in humans. Numerous immune cells will infiltrate the site of infection and pro-inflammatory cytokines will be released in an effort to eliminate the virus, but this response is largely immunopathologic; leading to swelling, tissue damage and chronic arthralgia (47, 48). After the initial infection, long-term protection against alphavirus-induced disease, including foot swelling in mice, is thought to be mediated mainly by neutralizing antibodies (41, 49). In our study, ChAdOx1 May prevented MAYV-induced foot swelling and also delayed and diminished the swelling caused by CHIKV. In contrast, ChAdOx1 Chik vaccination did not prevent the foot swelling caused by MAYV. Another study (25), reported that vaccination of A129 mice with an insect-specific virus platform containing CHIKV structural proteins did not afford protection and resulted in increased foot swelling.

Neutralizing antibodies are a key correlate of protection against alphaviruses. Cross-neutralization is likely to occur because MAYV and CHIKV are phylogenetically related, with the strains used in this study sharing approximately 71% of amino acid similarity. Webb and colleagues (25), reported cross-protection against MAYV disease in A129 mice vaccinated with a live attenuated CHIKV-IRES vaccine, with this phenomenon demonstrated to be antibody mediated and not altered by T cell responses. In support, a selected group of monoclonal antibodies generated from CHIKV-infected mice were shown to be broadly neutralizing, capable of limiting the viral lifecycle of several alphaviruses by blocking their cell entry and exit (22). Authors performing PRNT assays using convalescent human sera from CHIKV infected individuals, have also described a degree of cross-neutralizing activity against MAYV (24, 25). However, a recent publication (50) reports that equally potent monoclonal neutralizing antibodies against the same MAYV epitope, some of which were even also cross-reactive against CHIKV, did not protect mice from MAYV disease equivalently. They were able to find that the effectiveness of their antibodies was not only related to the neutralization potency, but it was also related to the antibody*’*s F_c_ effector function on phagocytosis and cytolysis. Although in this work ChAdOx1 Chik and ChAdOx1 May afforded significant cross-protection against heterologous disease, we did not detect significant titers of cross-neutralizing antibodies.

Antibodies against E2 protein, in particular those binding to the β domain, appear to be strongly neutralizing (22), although there is evidence suggesting that neutralization against E1 protein may also be important (22). We detected specific IgG antibody binding to MAYV E2 and E1 and CHIKV E2 and E1 as early as 2 weeks after immunization with ChAdOx1 May and ChAdOx1 Chik, respectively. Some degree of cross-reactivty was observed in both ChAdOx1 May and ChAdOx1 Chik vaccinated groups, in particular toward CHIKV E2 and MAYV E2 proteins. This induced cross-reactive anti-E2 antibodies may partially account for degree of cross-protection observed in our A129 challenge model.

In summary, we provide evidence of the protective efficacy of ChAdOx1 May against MAYV, as well as its cross-reactive effects on CHIKV. If this cross-protection also takes place in the context of immunity secondary to natural infection, it is likely that the emergence potential of MAYV may be reduced by pre-existing CHIKV immunity. Reciprocally, immunity to MAYV may affect the breadth of the ongoing CHIKV outbreaks, modulating both geographical spread and the severity of the impact on human health. Overall, our work sheds light into the immunogenic interactions between MAYV and CHIKV and is of high relevance in the occurrence of large outbreaks in areas where CHIKV and MAYV co-circulate. Finally, it is also important for the development of Phase II/III clinical trials that aim to assess the efficacy of CHIKV and MAYV vaccine candidates in endemic areas where cross-reactive pre-existing immunity may be present. In the future, a dual vaccination approach with ChAdOx1 May and ChAdOx1 Chik should be tested, as there may be the possibility of immune synergy to occur.

## Data Availability Statement

The raw data supporting the conclusions of this article will be made available by the authors, without undue reservation.

## Ethics Statement

Animal manipulations were done according to an approved Institutional Animal Care and Use (IACUC) protocol (1708051).

## Author Contributions

Conceptualization: RC, LP-L, AR-S, SR, CL-C, and SA. Formal analysis: RC, LP-L, and SR. Investigation, RC, SA, YK, and SR. Resources: LP-L, YK, CL-C, AR-S, and SR. Data curation: RC and SR. Writing—original draft preparation: RC, LP-L. Writing—review and editing: RC, LP-L, YK, SA, AR-S, and SR. Project administration: AR-S and SR. All authors contributed to the article and approved the submitted version.

## Funding

This research was funded by Innovate UK (reference number 971557) with funds supplied by the United Kingdom Department for Health and Social Care Innovate UK.

## Conflict of Interest

The authors declare that the research was conducted in the absence of any commercial or financial relationships that could be construed as a potential conflict of interest.
